# Mannose-Binding Lectin Inhibits Monocyte Proliferation through Transforming Growth Factor-β1 and p38 Signaling Pathways

**DOI:** 10.1371/journal.pone.0072505

**Published:** 2013-09-06

**Authors:** Yan Wang, A-De Chen, Yan-Mei Lei, Gui-Qiu Shan, Li-Yun Zhang, Xiao Lu, Zheng-Liang Chen

**Affiliations:** 1 Department of Immunology, Southern Medical University, Guangzhou, Guangdong, China; 2 Department of Microbiology and Immunology, School of Basic Medicine, Guangdong Medical College, Dongguan, Guangdong, China; St. Jude Children's Research Hospital, United States of America

## Abstract

Mannose-binding lectin (MBL), a plasma C-type lectin, plays an important role in innate immunity. However, the interaction, and the consequences of it, between MBL and the immune system remain ill defined. We have investigated the contributing mechanisms and effects of MBL on the proliferation of human monocytes. At lower concentrations (≤4 μg/ml) MBL was shown to partially enhance monocyte proliferation. By contrast, at higher concentrations (8–20 μg/ml) of MBL, cell proliferation was markedly attenuated. MBL-induced growth inhibition was associated with G0/G1 arrest, down-regulation of cyclin D1/D3, cyclin-dependent kinase (Cdk) 2/Cdk4 and up-regulation of the Cdk inhibitory protein Cip1/p21. Additionally, MBL induced apoptosis, and did so through caspase-3 activation and poly ADP-ribose polymerase (PARP) cleavage. Moreover, transforming growth factor (TGF)-β1 levels increased in the supernatants of MBL-stimulated monocyte cultures. We also found that MBL-dependent inhibition of monocyte proliferation could be reversed by the TGF-β receptor antagonist SB-431542, or by anti-TGF-β1 antibody, or by the mitogen-activated protein kinase (MAPK) inhibitors specific for p38 (SB203580), but not ERK (U0126) or JNK (SP600125). Thus, at high concentrations, MBL can affect the immune system by inhibiting monocyte proliferation, which suggests that MBL may exhibit anti-inflammatory effects.

## Introduction

The innate immune system recognizes and rapidly responds to microbial pathogens, and in doing so provides a first line of host defense. A defective innate immune system can increase the host's susceptibility to infection. In addition, dysregulation of innate immunity is seen in many diseases and may contribute to Alzheimer's disease [1], development of tumors, and autoimmune disease, among others. Dysregulated immunity may also contribute to chronic inflammatory conditions in the human populations, including Crohn's disease [2].

Monocytes and macrophages are an essential component of the innate immune system, and possess a multitude of immunological functions, including phagocytosis and endocytosis, cytokine production and antigen presentation. Additionally, the capacity of monocytes to initiate inflammation and recruit other immune cells is complemented by their ability to present antigens in the context of products of the major histocompatibility complex (MHC), making them an important link between the innate and adaptive immune systems.

A balanced network of cell survival and death proteins determines the fate of monocytes. Molecular interactions occurring during early G1 cell cycle arrest, may be important in determining cell fate [3]. The presence of stimulatory signals triggers monocyte survival by inhibiting the apoptotic pathway, thus contributing to the maintenance of the inflammatory response [4]. Subsequently, as inflammation resolves, the apoptotic program resumes, and monocytes undergo apoptosis, which facilitates the resolution of an immune response [4].

Mannose-binding lectin (MBL), is a member of the collectin family of the C-type lectin superfamily, and is a multimeric protein containing collagen-like sequences. MBL is synthesized and secreted into the blood by hepatocytes. Thus far, serum-borne MBL has been intensively characterized and found to behave as a key pattern recognition molecule, which recognizes carbohydrates on the surface of microbial pathogens [5]. Following pathogen recognition, MBL may activate the complement cascade through the lectin pathway, after which microbes are targeted for cellular lysis and indirect opsonization. When binding to the collectin receptor of effector cells, MBL mediates direct opsonization and cell-mediated cytotoxicity [6]. MBL also augments the phagocytosis of cellular debris, apoptotic cells and immune complexes both *in vitro* and *in vivo* [7]. As such, MBL is considered a key molecule in innate immunity.

Recently, it has been demonstrated that the innate immune response markedly influences and determines the nature of the subsequent adaptive immune response [8]. It is clear that the innate immune system activates appropriate adaptive immune responses via ligand interactions and secretion of cytokines and chemokines [9]. For instance, interaction of MBL with phagocytes alters subsequent phagocyte cytokine synthesis, which may have important implications in directing acute inflammation, and long-term protective immunity [10].

MBL has been reported to regulate dendritic cell (DC) maturation, and cytokine production on activation by bacterial lipopolysaccharide (LPS) [11], and may influence the cytokine network after stimulation by various microorganisms [12]. Stimulation of mature DCs by culturing them in the presence of recombinant human MBL (rhMBL) and subsequently co-culturing the MBL-exposed DCs with allogeneic mononuclear cells, markedly promoted the secretion of interleukin (IL)-1β, IL-6, and tumor necrosis factor (TNF)-α *in vitro* [13]. In most co-cultures of DC and mononuclear cells, the secretion of interferon (IFN)-γ was also enhanced. In this context, it is interesting to note that MBL can bind to human monocytes *in vitro* and that such interactions are calcium-dependent and highly specific. We speculate that such interactions can exert important effects on peripheral blood monocytes. We therefore aimed to investigate whether MBL could influence the proliferation of human monocytes. Furthermore, we aimed to determine the molecular mechanisms underlying the interactions of MBL and monocytes.

## Materials and Methods

### Preparation of MBL

MBL was isolated from human plasma according to the method published by Tan et al. [14], and modified as described [15]. In brief, thawed pooled human plasma was treated to extract and eliminate most of the unrelated proteins, and the remainder was solubilized. MBL was subsequently purified from the processed extract by three successive chromatographic steps. The first step was affinity chromatography on a mannan-agarose column (Sigma, Poole, UK), to select for functionally active, carbohydrate-binding MBL with an approximate 2000-fold purification. Subsequent purification steps utilized anion-exchange chromatography and gel filtration coupled with a Mono-Q HR 5/5 column (Pharmacia Biotech Europe, Orsay, France) and a Superose 6 HR 10/30 column (Pharmacia Biotech Europe). Purified MBL was identified by sodium dodecyl sulfate-polyacrylamide gel electrophoresis (SDS-PAGE) and Western immunoblot (Figure. S1). MBL preparations were not contaminated with endotoxin as determined by the Limulus amebocyte lysate assay.

### Cell culture

Monocytes were purified from peripheral blood mononuclear cells (PBMCs) prepared from peripheral blood donations. Briefly, PBMCs were isolated by density gradient centrifugation over Ficoll as previously described [16] and monocytes were purified from the PBMC by immuno- magnetic negative selection using the human Monocyte Isolation Kit II (Miltenyi Biotec, Germany). Monocytes or the human monocytic U937 cell-line (a gift from the Cancer Research Center, Sun Yat-Sen University, Guangzhou, China) were maintained in endotoxin-free RPMI-1640 (Gibco BRL, Gaithersburg, MD, USA), and supplemented with 10% (v/v) heat-inactivated fetal calf serum (Gibco BRL, Grand Island, CA, USA) and cultured at 37°C in a 5% (v/v) CO_2_ in air fully-humidified incubator.

### Analysis of MBL binding to monocytes

Washed monocytes, and at a density of 1×10^6^ cells/ml were resuspended in Tris-buffered saline, pH 7.4, supplemented with 10 mM CaCl_2_ and 1% bovine serum albumin. Each cell suspension (0.2 ml, 2×10^5^) was incubated for 30 min at 37°C in the presence of the indicated concentrations of MBL, which had been previously labeled with fluorescein isothiocyanate isomer 1 (FITC; Sigma, Poole, UK). Three kinds of Tris-buffered saline, which contained varying concentrations of calcium was alternatively used for the binding assays. In the Ca^2+^-free control, 5 mM ethylenediaminetetraacetic acid (EDTA) was substituted for CaCl_2_. Moreover, monocytes were preincubated with or without unlabelled-MBL (200 μg/ml) for 30 min, and then incubated with 4 μg/ml of FITC-MBL for 30 min. After washing, the binding of MBL to the cells was analyzed by flow cytometry (FCM) using a FACSCalibur (Becton Dickinson, Mountain View, CA, USA).

### Proliferation assays

50 ng/ml recombinant human macrophage colony stimulating factor (rhM-CSF, Peprotech, Rocky Hill, USA)-induced monocytes were seeded into 96-well plates (Nunc 167008, Nunclon, Raskilde, Denmark) at 2.5×10^4^ cells/well in triplicate. U937 cells were seeded into 96-well plates at 5×10^3^ cells/well in triplicate. Different concentrations of MBL were added to each well (except controls) and the plates were incubated at 37°C for 24, 48 and 72 h. On the time indicated, cell viability was assessed by using the Cell Counting Kit-8 (CCK-8; Dojindo Laboratories, Kumamoto, Japan) and according to the manufacturer's instructions. In some experiments, monocytes were incubated with MBL at 20 μg/ml alone or with 0.2 μg/ml affinity-purified, neutralizing rabbit anti-macrophage colon-stimulating factor (M-CSF) (Abcam, Cambridge, UK) or with 0.1 μg/ml anti-transforming growth factor-β1 (TGF-β1) (Abcam, Cambridge, UK) or in the presence of the TGF-β1 receptor antagonist SB-431542 (5 μM, Abcam, Cambridge, UK). Occasionally a range of chemical inhibitors (LY294002, U0126, SB203580, SP600125, Cell Signaling Technology, Beverly, USA) were added to each well (except controls) and plates were incubated at 37°C for 0.5 h. Whereafter cells were treated by MBL at 20 μg/ml and further incubated at 37°C for 24, 48 and 72 h.

For testifying our results of the CCK-8 assay, we further performed another method of thymidine incorporation assay to quantify more precisely the real % of proliferating monocytes in-vitro in the presence or absence of MBL. 50 ng/ml rhM-CSF-induced monocytes were seeded into 96-well flat-bottomed plates at 2×10^5^ cells/well in triplicate. Different concentrations of MBL were added to each well (except controls). All cultures were prolonged at 37°C for 24, 48 and 72 h, including an overnight incubation in the presence of ^3^H- thymidine (1 μCi/well, Amersham, Little Chalfont, UK). Cultures were then harvested and incorporated radioactivity was measured by liquid scintillation counting. The experiences were performed more than three times.

### Cell cycle analysis

Washed U937 cells were treated with various concentrations of MBL for 12, 24, and 48 h, respectively. The cells were collected and fixed in 70% alcohol overnight at −20°C, following which, cells were stained with propidium iodide (PI) staining buffer (Trixon X-100, EDTA, RNase A, PI) for 30 min at 4°C in the dark. A FACSCalibur flow cytometer was used to monitor the fluorescence of PI (50 μg/ml; Sigma, USA). Cell cycle distributions were analyzed by flow cytometry using the ModFit LT software program for Win32 (Verity Software House, ME, USA).

### Analysis of nuclear morphology

U937 cells were fixed in 4% paraformaldehyde at 4°C for 10 min and stained with Hoechst 33258 at a concentration of 1 mg/ml, and at 37°C in the dark for 30 min. The cells were washed three times in PBS, examined, and immediately photographed under a fluorescence microscope (Olympus, Japan) with an excitation wavelength of 330–380 nm.

### DNA laddering detection

U937 cells, at a density of 2×10^5^ cells/well, were treated with 20 μg/ml MBL for 72 h, collected and centrifuged at 1500 g for 5 min. DNA was extracted and assayed using the Apoptosis DNA Ladder Detection Kit (KeyGen Biotech Company, Nanjing, China) according to the manufacturer's instructions. The pattern of DNA fragmentation was assessed by agarose gel electrophoresis.

### Annexin V-FITC and PI double staining

Apoptotic cells were counted using the Annexin V-FITC Apoptosis Detection Kit (KeyGen Biotech Company, Nanjing, China) according to the manufacturer's instructions. Each sample was analyzed promptly and within 1h using a FACSCalibur flow cytometer and FCS Express V3 Software (De Novo Software, Canada), and the total apoptosis rate was calculated.

### Real-time RT-PCR analysis

Total cellular RNA was isolated using TRIZOL reagent (Invitrogen, Carlsbad, CA). The M-MLV First-Strand Synthesis System (Invitrogen, Carlsbad, CA) for real-time PCR was used to synthesize first-strand cDNA from total RNA. The primer sequences are shown in Table. S1. Quantitative real-time PCR was performed in the Rotor-Gene 6000 real-time PCR detection system (Rotor-Gene 6000; Qiagen, Germany). Reactions were completed in a 20 μl volume containing a mixture of cDNA, specific primers of each gene, and the platinum SYBR green supermix-UDG (Invitrogen, Carlsbad, CA). The cycling conditions were 50°C for 2 min, 95°C for 2 min, followed by 40 cycles of 95°C for 15 s and 60°C for 60 s. The specific PCR products were determined by measuring the fluorescence of SYBR Green, which stained double stranded DNA. The relative mRNA expression levels were calculated from the threshold cycle (Ct) value of each PCR product, and normalized against GAPDH using the comparative Ct method. The relative expression of each gene of untreated control cells was set to 1, and that of MBL-treated cells was then converted to a relative fold change in expression level relative to untreated control cells.

### Assessment of intracellular caspase-3 activity

The CaspGlow^TM^ Fluorescein Active Caspase-3 Staining Kit (BioVision, Mountain View, CA, USA) was used to detect the activity of intracellular caspase-3 following the manufacturer's instructions.

### Western immunoblot analysis

U937 cells were treated with and without MBL for 72 h, then harvested and lysed in ice-cold lysis buffer (1 M Tris-HCl, 5 M NaCl, 1% Nonidet P-40 (v/v), 1% sodium deoxycholate, 0.05% sodium dodecyl sulfate, 1 mM phenylmethylsulfonyl fluoride) for 20 min at 4°C. After brief sonication the lysates were centrifuged at 12, 000×*g* for 15 min at 4°C, and the protein content in the supernatant was quantitated by the Bicinchoninic acid (BCA) method (Santa Cruz, San Diego, CA, USA). Next, 50 μg of total protein was subjected to SDS-PAGE in 8–15% polyacrylamide gels and transferred onto polyvinylidene difluoride (PVDF) membranes (Immobilon-P^SQ^ transfer membrane, Millipore, USA). Membranes were incubated with blocking buffer (5% non-fat dry milk in PBS containing 0.1% Tween-20) overnight at 4°C. Western immunoblot analysis was carried out to detect the expression of specific proteins using specific anti-caspase-3 polyclonal antibody (pAb), anti-poly ADP-ribose polymerase (PARP) monoclonal antibody (mAb), anti-Fas mAb, anti-FasL mAb, and anti-GAPDH mAb (Santa Cruz at a dilution of 1∶1000). The membrane was incubated with the indicated primary antibodies for 2 h at room temperature, and then treated with the appropriate horseradish peroxidase conjugated anti-mouse IgG or peroxidase conjugated anti-rabbit IgG (Santa Cruz at a dilution of 1∶1000) for 2 h at room temperature, and the immunoreactive bands were detected by enhanced chemiluminescence (ECL) reagent (Amersham Biosciences, Amersham, UK). The intensity of the protein bands was quantified using Quantity-One software and the ratio of the specific bands to the control was subsequently analyzed.

### Measurement of TGF-β1 by ELISA

Following stimulation of human monocytes, the culture supernatants were harvested, following which ELISA was used to determine the concentrations of TGF-β1 (eBioscience, San Diego, CA, USA) in accord with the manufacturer's instruction.

### Statistical analysis

Data were analyzed using the SPSS 13.0 statistical software for Windows (SPSS Inc., Chicago, IL, USA). Results were expressed as the mean ± standard deviation (SD). Statistical significance between groups was analyzed by one-way ANOVA, and multiple comparisons of data were performed by the Bonferroni correction test, or Dunnett T3's test. A P-value of <0.05 was considered statistically significant.

## Results

### MBL binds to monocytes

Functional MBL receptor expression by many cell types remains controversial. As a major soluble pattern-recognition receptor (PRR) in the innate immune system, MBL has long been known to recognize pathogens or autologous apoptotic cells via its carbohydrate recognition domain (CRD), and to interact with normal autologous cells via its collagen-like region (CLR). It was believed that MBL-CRD does not interact with normal autologous cells. However, Downing et al. [16] reported calcium-dependent MBL binding to autologous B lymphocytes, monocytes and immature monocyte-derived DCs via its C-type lectin-binding site, which suggested a new role for MBL in the immune system.

**Figure 1:**
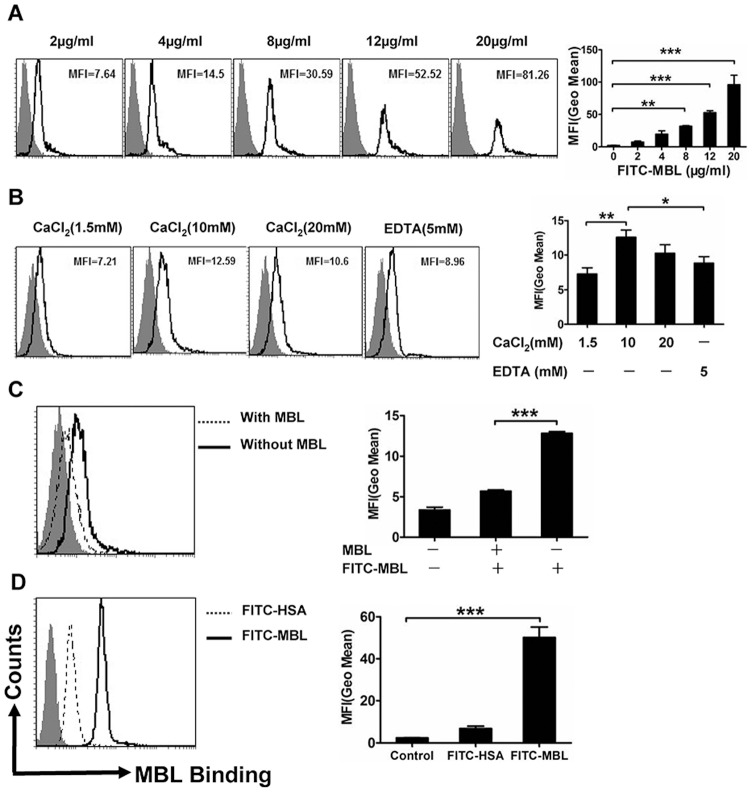
Analysis of binding of MBL to monocytes by FCM. Monocytes were treated *in vitro* with various concentrations of FITC-MBL (A). The shaded curves describe cells only; the black solid line shows FITC-MBL. Ca^2+^-dependent binding of MBL to monocytes (B), where monocytes were pretreated *in vitro* with tris-buffer saline, and then treated with 4 μg/ml of FITC-MBL. (C) Shows that unlabelled-MBL attenuated the binding of FITC-MBL to monocytes. Monocytes were preincubated with (dotted line) or without (black solid line) unlabelled-MBL (200 μg/ml), and further incubated *in vitro* with 4 μg/ml of FITC-MBL. (D) Shows MBL binding to U937 cells, where U937 cells were treated with 10 μg/ml of FITC-human serum albumin (HSA) (dotted line) or 10 μg/ml of FITC-MBL (black solid line). These data are the representatives of three independent experiments. Each bar represents the mean ± S.D. Data were evaluated by one-way ANOVA and multiple comparisons were performed by Bonferroni or Dunnett T3's test. * P<0.05, ** P<0.01, *** p<0.001.

In this study, the ability of MBL to bind to monocytes and U937 cells was determined by FCM. Representative flow histograms depicting the binding of various concentrations of FITC-MBL to monocytes are shown (Figure. 1A). Of particular note, we found that binding was markedly increased by stimulation with 10 mM Ca^2+^. In addition, the binding was markedly reduced in the absence of Ca^2+^. These results showed that MBL interacted with monocytes in a Ca^2+^-dependent manner (Figure. 1B). The binding was optimal at supra-physiological concentrations, which suggested that MBL receptors or binding proteins may exist on the surface of monocytes [17]. Additionally, following preincubation of the cells with excessive unlabelled-MBL (200 μg/ml), the cell-surface binding of FITC-MBL (4 μg/ml) was significantly attenuated, which suggested specificity in the binding of MBL to monocytes (Figure. 1C). We found that MBL directly bound to U937 cells (Figure. 1D). This data is comparable with findings from our previous studies [11], [18].

### MBL regulates proliferation of monocytes

**Figure 2:**
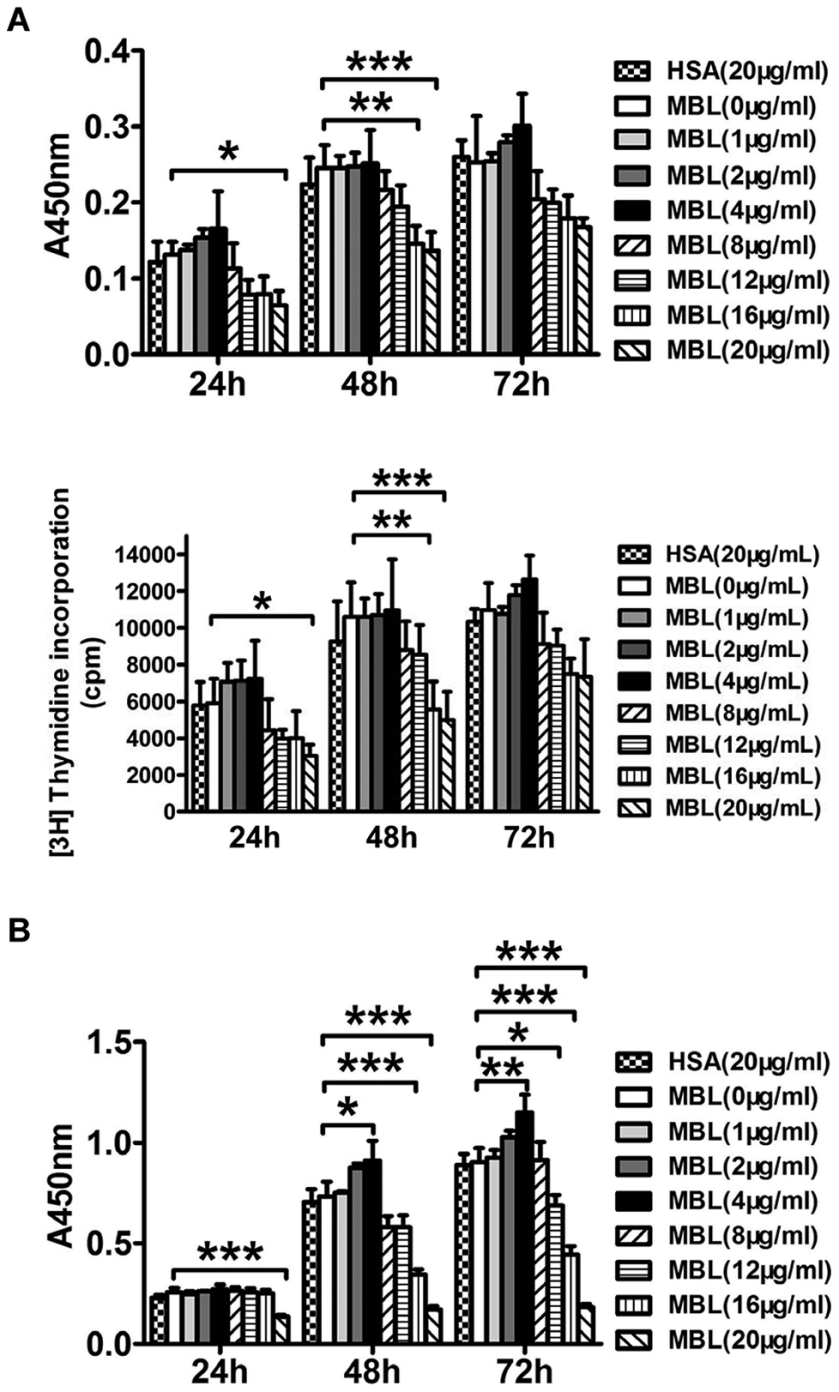
MBL regulates monocyte proliferation. Effects of MBL on proliferation of monocytes (A) stimulated with 50 ng/ml rhM-CSF were assessed by the CCK-8 assay and the thymidine incorporation assay. Effects of MBL on proliferation of U937 cells (B) were assessed by the CCK-8 assay. Data are described as mean ± S.D. of three independent experiments. * P<0.05, ** P<0.01, *** P<0.001 as compared with the 0 μg/ml MBL group.

In this study, we found a significant association between proliferative response and levels of MBL. MBL regulated cell proliferation in a concentration and time-dependent manner (Figure. 2A and 2B). Treating U937 cells with a lower concentration of MBL (≤4 μg/ml) resulted in increased proliferation as determined by the CCK-8 assay following 48 h and 72 h of treatment (p = 0.033, p = 0.002). However, a significant decrease in cellular proliferation was seen when U937 cells were treated with higher concentrations of MBL (8–20 μg/ml) for 24 h, 48 h and 72 h (p = 0.000, p = 0.01). Moreover by the CCK-8 assay and the thymidine incorporation assay we also found that similarly higher concentrations of MBL (8–20 μg/ml) could significantly decrease proliferation when monocytes were treated at 24 h and 48 h (p = 0.036, p = 0.001, p = 0.000), although there was a non-statistically significant decrease at 72 h. Namely, MBL has a double-edged role in immunity, and the presence of MBL could be either a mitigating or an aggravating factor in monocyte proliferation.

### MBL induces cell arrest in the G0/G1 phase of the cell-cycle

**Figure 3:**
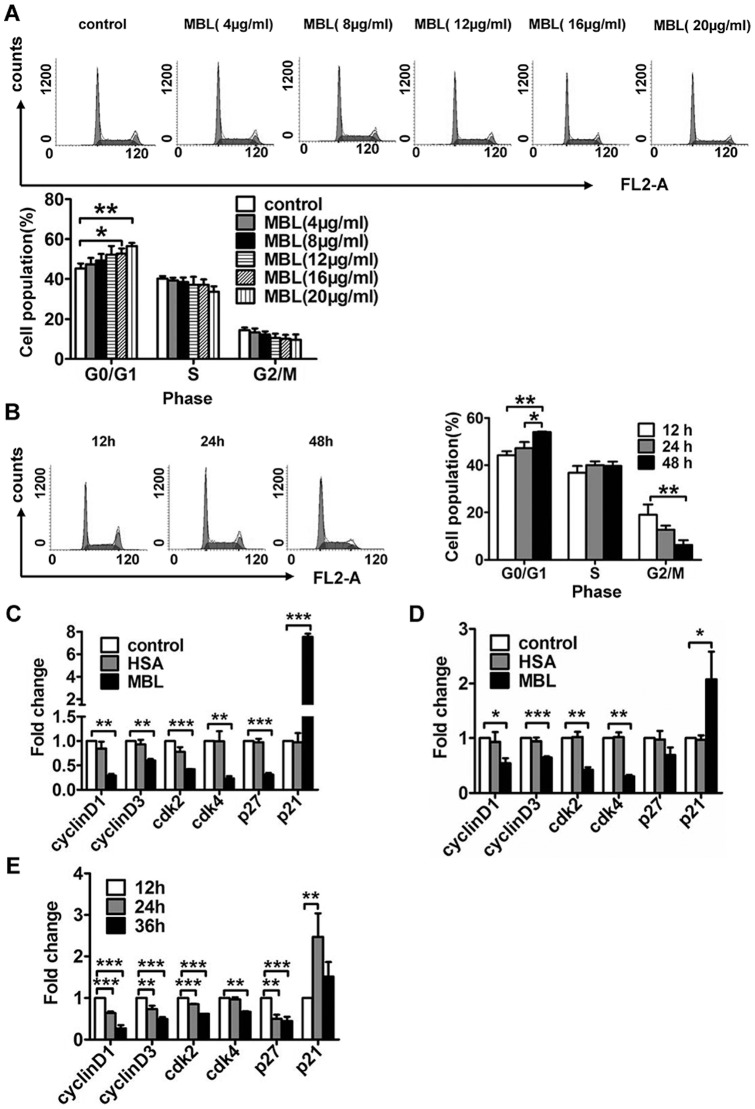
Regulation of monocyte cell cycle by MBL. (A and B) Cell cycle distribution of U937 cells treated with MBL. FCM analysis of the cell cycle distribution induced by MBL for 24 h at the indicated concentrations (A) and by 8 μg/ml MBL for the indicated times (B). (C–E) The effect of MBL on the mRNA expression of cell cycle regulatory proteins was analyzed by real-time RT-PCR. (C) U937 cells, and (D) monocytes were treated with vehicle, HSA (20 μg/ml) and MBL (20 μg/ml) for 24 h. (E) U937 cells were treated with MBL (8 μg/ml) for different times as indicated. Each bar represents the mean ± S.D. of three independent experiments. * p<0.05, ** p<0.01, *** p <0.001 as compared with the control cultures.

To explore the mechanism potentially responsible for the MBL-induced anti-proliferative effect, we investigated whether MBL induces cell arrest in human monocytes. Representative flow histograms depicting cell cycle distribution showed that exposure of U937 cells to MBL resulted in the enrichment of the G0/G1 phase, which was accompanied by a decrease in the G2/M phase (Figure. 3A and 3B). In addition, treatment of U937 cells with MBL for 24 h resulted in a significantly higher number of cells in the G0/G1 phase at the concentrations used, 16 μg/ml (52.790±2.589 %, p = 0.039) and 20 μg/ml (56.627±1.510%, p = 0.003), compared with vehicle-treated controls (45.270±2.485%) as shown in Figure. 3A. Here, we studied the effect of MBL on the cell cycle of monocytes, and further investigated the molecular mechanism.

### MBL regulates mRNA expressions of cell cycle regulating proteins

The cell cycle, or cell-division cycle, is the series of events that takes place in a eukaryotic cell, which leads to cellular replication [19]. Two key classes of regulatory molecules, referred to as cyclins and Cdks, determine the progress of a cell through the cell cycle [20]. Cyclin D/Cdk4 complexes are involved in the regulation of early G1, whereas the cyclin E/Cdk2 complex is required for the completion of G1 and initiation of the S phase of the cell cycle [21]. Cyclin/Cdk complexes can be inhibited by their interactions with cyclin-dependent kinase inhibitors (Cdki) such as Cip1/p21 and Kip1/p27.

Thus, we examined the effects of MBL on mRNA expression of cycle regulatory proteins in both monocytes and U937 cells. We found that MBL can induce G0/G1 arrest in monocytes, and U937 cells, and does so in both a dose- and time-dependent manner by increasing Cip1/p21 expression and dampening the expression of cyclin D1/ D3, Cdk2 and Cdk4 proteins (Figure. 3C–3E). Both Cip1/p21 and Kip1/p27 are important factors in regulating the cycle, and they play an important role in both apoptosis and cell cycle arrest [22]. However, our data showed that MBL treatment reduced the expression of Kip1/p27 in U937 cells. The reason was unclear, but it might be that Kip1/p27 didn't play a major role in cell cycle arrest induced by MBL. Thus it could be seen that MBL-induced G0/G1 arrest in monocytes, and U937 cells is also mediated through the up-regulation of Cip1/p21. This enhances the formation of heterotrimeric complexes with G1-S Cdks and cyclins, and thereby inhibiting their activity. There was evidence that deficiency of p21 or p27 had a different impact on the appearance of tumors and overall survival of the INK4a/ARF-null mice [23]. The absence of p27 in combination with mutant INK4a/ARF resulted in a significant acceleration of tumor development and progression that culminated in decreased survival. In contrast, p21 deficiency in the context of INK4a/ARF-null mice had a modest effect on tumorigenesis and survival [23].

### MBL induces apoptosis

**Figure 4:**
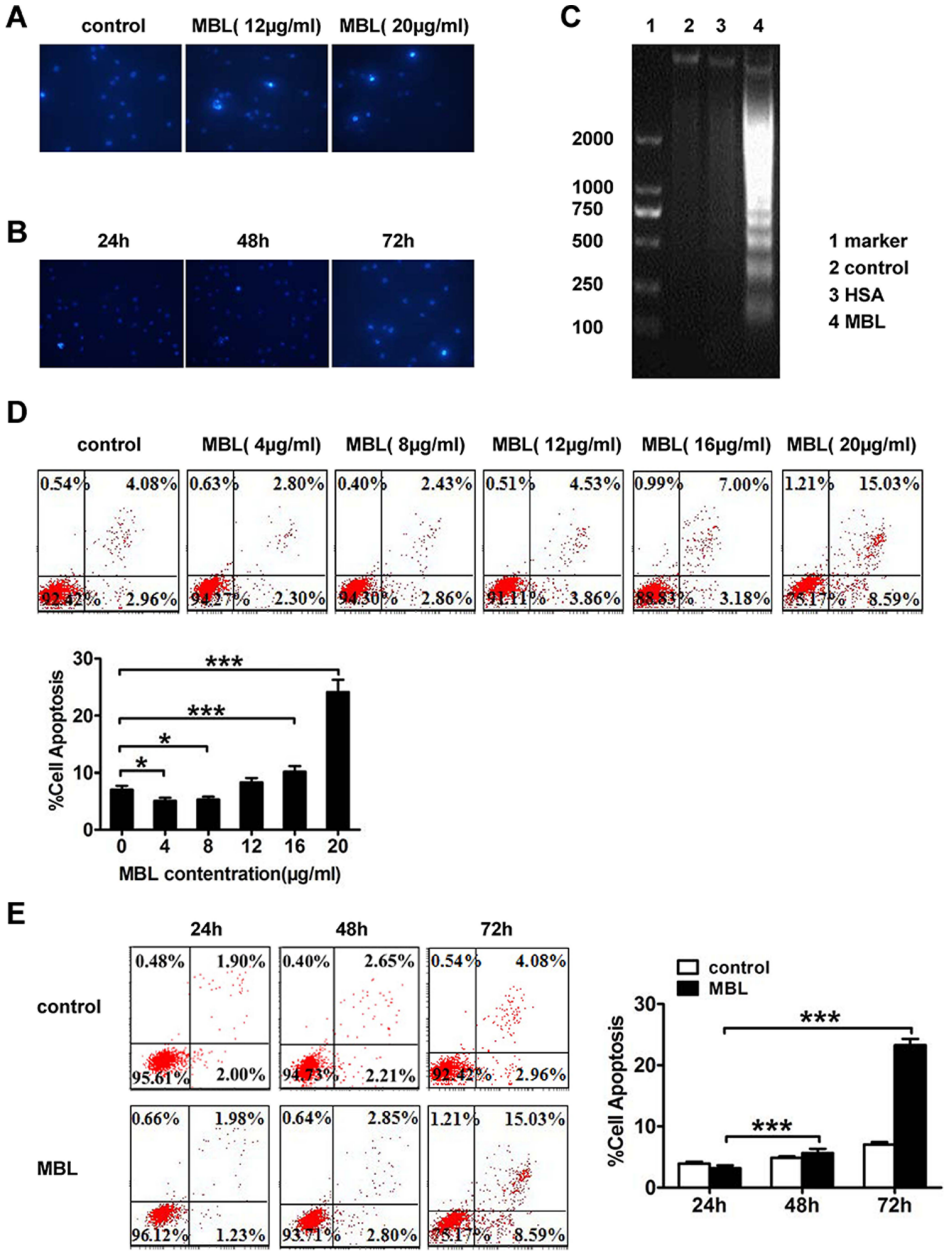
Induction of apoptosis of U937 cells following treatment with MBL. Morphological changes in the nuclei (typical of apoptosis) of cultured U937 cells (A and B). U937 cells were treated with various concentrations of MBL (A) and following different treatment times (B), and then stained with Hoechst 33258 and viewed under a fluorescence microscope (×40). We found that 20 μg/ml of MBL induced DNA fragmentation in U937 cells, as determined by agarose gel electrophoresis (C). Lane 1: DNA marker; Lane 2: control cells; Lane 3: 20 μg/ml of HSA; Lane 4: 20 μg/ml of MBL. The induction of apoptosis was determined by Annexin V-FITC/PI double-staining assay (D and E). (D) Shows a dose-dependent effect of MBL in U937 cells. The cells were treated with 0, 4, 8, 12, 16 and 20 μg/ml of MBL for 72 h. (E) Shows the time-dependent increases in apoptotic cells in U937 cells. U937 cells were treated with 20 μg/ml of MBL or vehicle for 24 h, 48 h, and 72 h. The figures were the representatives of three separate experiments. Each bar represents the mean ± S.D. * P<0.05, ** P<0.01, *** p<0.001 show levels of statistical significance as compared with 0 μg/ml or resting conditions (D) or from cells treated for 24 h (E), as measured by Dunnett's test.

To explore the mechanism potentially responsible for the MBL-induced anti-proliferative effect, we investigated whether MBL induces apoptosis in human monocytes. Hoechst 33258 fluorescence photomicrographs of cultured U937 cells treated with 0, 12, and 20 μg/ml of MBL for 72 h, respectively (Figure. 4A). In control cultures, the nuclei of U937 cells appeared with regular contours, and were round and large in size. Rarely, we saw U937 cells that showed smaller nuclei and condensed chromatin. Treatment of cells with MBL at 20 μg/ml for either 24 h or 48 h did not lead to morphologic changes of the nuclei (Figure. 4B). In contrast, most nuclei isolated from U937 cells treated with 20 μg/ml MBL appeared hypercondensed (brightly stained) in 72 h. Agarose gel electrophoresis of soluble DNA from MBL-treated U937 cells revealed DNA fragmentation, characteristic of apoptotic cells (DNA ladder). However, DNA fragmentation was not observed in U937 cells after 72 h of culture in the absence of MBL treatment. The results indicated that the exposure of U937 cells to 20 μg/ml of MBL led to DNA fragmentation within 72 h of treatment (Figure. 4C). U937 cells were treated with MBL at 20 μg/ml for 72 h to study apoptosis following staining with Annexin V-FITC and PI (Figure. 4D and 4E). We found that untreated control cells (7.058±0.688 %) were positive for Annexin V-FITC while cells (24.147±2.151 %) were positive for Annexin V-FITC following treatment with MBL at 20 μg/ml, and showed a marked increase in apoptosis (p = 0.000).

### MBL regulates the expressions of apoptosis regulators

**Figure 5:**
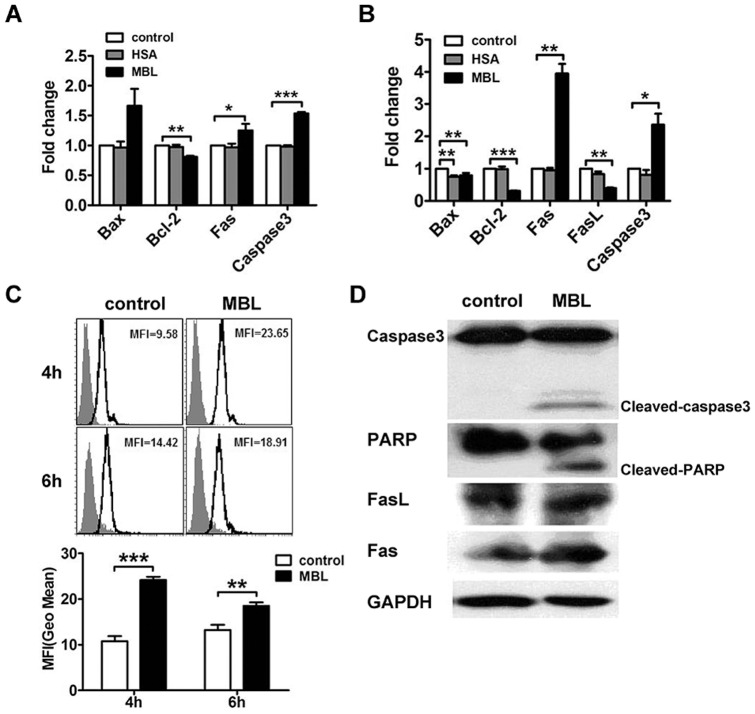
The effects of MBL on the expressions of apoptosis regulators in monocytes. The effects of MBL on the mRNA expression of caspase-3, Fas, FasL, Bax, and Bcl-2 as shown by real-time RT-PCR (A and B). Monocytes were treated with vehicle, HSA (20 μg/ml) and MBL (20 μg/ml) for 48 h (A). U937 cells were treated with vehicle, HSA (20 μg/ml) and MBL (20 μg/ml) for 72 h (B). MBL induced caspase-3 activity (C). U937 cells were treated with 20 μg/ml of MBL or vehicle for 4 h and 6 h. The activity of caspase-3 was determined by FCM. The effects of MBL on caspase-3, PARP, Fas and FasL protein expression, as determined by Western immunoblotting (D). U937 cells were treated with MBL at 20 μg/ml or vehicle for 72 h. Caspase-3 activation and PARP cleavage were detected by Western blotting. GAPDH was used as the internal control. Data were mean ± S.D. of three independent experiments. Levels of statistical significance were: * p<0.05, ** p<0.01, *** p<0.001 as compared control cultures.

The effects of MBL on apoptosis-related factors were further investigated. Analysis by real-time RT-PCR showed that MBL at 20 μg/ml significantly increased the mRNA expression of caspase-3 and Fas, and decreased the mRNA expression of Bcl-2 in monocytes and U937 cells (Figure. 5A and 5B). We also found that the level of pro-apoptotic Bax insignificantly increased in monocytes but significantly decreased in U937 cells, while induction of Fas was far greater in U937 cells than monocytes, suggesting that there were cell-type specific differences in the mechanisms of apoptosis induction by MBL. Western immunoblot analysis showed that MBL at 20 μg/ml enhanced the expression of Fas, and then sequentially activated caspase-3, which results in the cleavage of PARP consequently (Figure. 5D).

### MBL increases caspase-3 activity

To further validate that the effectors caspases are activated by MBL, we examined the activity of caspase-3 in U937 cells with or without MBL treatment. Our data showed that treating U937 cells with MBL at 20 μg/ml caused pronounced activation of caspase-3 as indicated by increased fluorescence intensity. These results indicated that the activity of caspase-3 increased between 4 h and 6 h of exposure following treatment with MBL at 20 μg/ml (Figure. 5C). Both 4 h and 6 h exposure showed similar kinetics in the occurrence of apoptosis [24].

### MBL induces secretion of TGF-β1 and reduces proliferation

**Figure 6:**
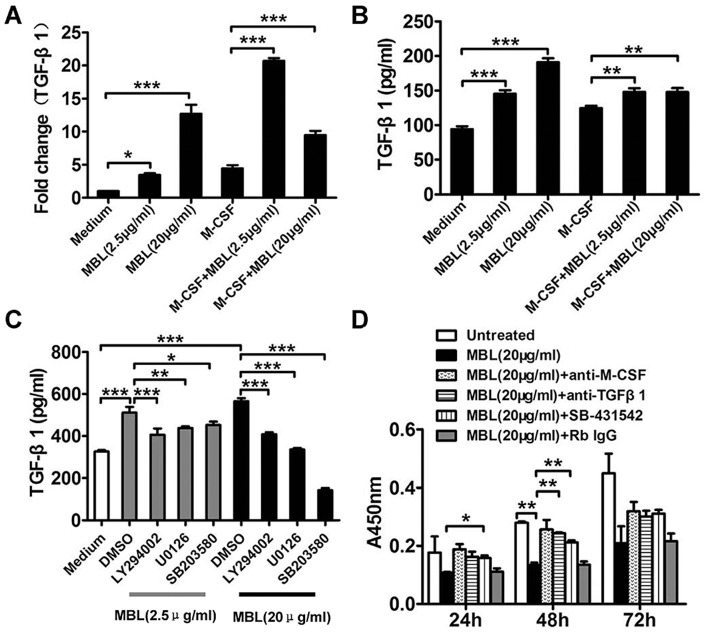
MBL inhibits monocytes proliferation by TGF-β1. Monocytes or 50 ng/ml rhM-CSF-induced monocytes were incubated with MBL at 2.5 μg/ml or 20 μg/ml for 24 h (A and B). MBL augmented TGF-β1 secretion. The effects of MBL on TGF-β1 mRNA expression were analyzed by real-time RT-PCR (A). The effects of MBL on the secretion of TGF-β1 were analyzed by ELISA (B). The expression of TGF-β1 induced by MBL was dependent on PI3K, ERK and p38 pathways (C). Monocytes were pretreated with 0.2% DMSO vehicle control or with inhibitors against PI3K (LY294002, 5 μM), ERK (U0126, 5 μM), and p38 (SB203580, 10 μM) at 37°C for 0.5 h, and then treated with MBL at 2.5 μg/ml or 20 μg/ml for 24 h. TGF-β1 expression levels were measured by ELISA. It was the addition of anti-TGF-β1 or the TGF-β1 receptor antagonist SB-431542 rather than control Rb IgG that abolished the inhibitory effect of MBL and restored the growth rate of monocytes to almost control levels (D). rhM-CSF-induced (50 ng/ml) monocytes were incubated with MBL alone at 20 μg/ml or in the presence of affinity-purified, neutralizing rabbit anti-M-CSF (0.2 μg/ml) or with anti-TGF-β1 (0.1 μg/ml) or with the TGF-β1 receptor antagonist SB-431542 (5 μM). Effects of MBL on proliferation of monocytes were assessed by the CCK-8 assay. Each bar represents the mean ± S.D. of three independent experiments. Indicated levels of significance are shown: * p<0.05; ** p<0.01; *** p<0.001.

Contact with or phagocytosis of apoptotic cells by macrophages causes the release TGF-β1 at levels of approximately 350 pg/ml [25]. The results of the current study indicated that MBL increased secretion of TGF-β1 by monocytes and TGF-β1 secretion by monocytes was more increased by high dose of MBL (20 μg/ml) than by low dose of MBL (2.5 μg/ml) (Figure. 6A and 6B). Such release and autocrine-like action of TGF-β1 on MBL-induced monocytes, promoted reduced proliferation, and such effects were also seen in our study. Moreover MBL also increased secretion of TGF-β1 by M-CSF-induced monocytes and the amplitude of TGF-β1 release by high dose of MBL in more differentiated monocytes (M-CSF treated) was less than in normal monocytes (Figure. 6A and 6B), suggesting that M-CSF seems to have a negative impact on high dose of MBL induced TGF-β1 secretion.

In order to explore the mechanisms of MBL, monocytes were preincubated with 0.2% DMSO as the vehicle control, or with inhibitors of PI3K (LY294002, 5 μM), ERK (U0126, 5 μM), and p38 (SB203580, 10 μM), following which, monocytes were incubated with MBL at 2.5 μg/ml or 20 μg/ml for 24 h. The secretion of TGF-β1 induced by MBL was shown to be dependent on PI3K, ERK and p38 pathways. Figure 6C explores the mechanisms of MBL-mediated TGF-β1 release.

This observation further implied that the addition of anti-TGF-β1 (0.1 μg/ml) or the TGF-β1 receptor antagonist SB-431542 (5 μM), could significantly abolish the inhibitory effect of MBL (20 μg/ml) but the addition of control rabbit IgG could not. In addition, treatment of monocytes with either anti-TGF-β1 (0.1 μg/ml) or the TGF-β1 receptor antagonist SB-431542 (5 μM), could restore the growth rate of 50 ng/ml rhM-CSF- induced monocytes to almost control level rates of proliferation (Figure. 6D). Inhibition TGF-β1 function in high MBL conditions could significantly increase the cell survival rate of monocytes at 48 h and it could result in an increased trend in the cell survival rate of monocytes at 72 h although there was a non-statistically significant increase at 72 h.

It is also quite well appreciated that active TGF-β1, mediated an immunosuppressive effects when interacting with the TGF-β receptor on effector cells. A few inhibitors of TGF-β1 signaling have been reported as potential therapeutics in cancer immunotherapy. Among these is the TGF-βR antagonist SB-431542, which is a selective inhibitor of endogenous activin, of TGF-β1 signaling [26], and the resulting phosphorylation of Smads. The tumor-inhibitory functions of TGF-β1 have also been reversed using SB-431542 in studies of colon cancer-derived FET cells. The antagonist has also been shown to suppress ligand-dependent growth of HT-29 colon cancer cells [27].

### MBL inhibits monocytes proliferation by p38-dependent pathway

**Figure 7:**
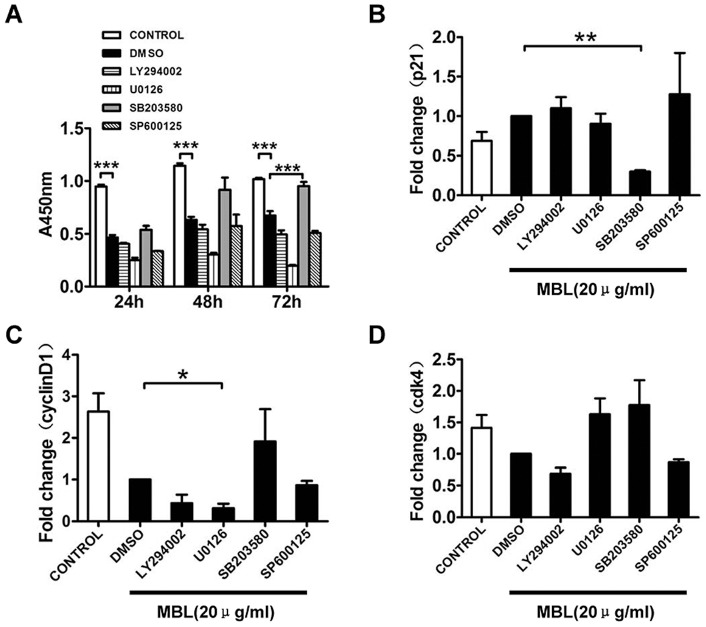
Inhibition of p38 significantly abolished the inhibitory effect of MBL on cellular proliferation. Monocytes were pretreated with 0.2% DMSO vehicle or inhibitors against PI3K (LY294002, 5 μM), ERK (U0126, 5 μM), p38 (SB203580, 10 μM), and JNK (SP600125, 5 μM) at 37°C for 0.5 h, and then treated with MBL at 20 μg/ml for 24 h, 48 h and 72 h. Cell proliferation rates were determined by CCK-8 assay (A). The expression levels of p21 (B), cyclinD1 (C), and Cdk4 (D) were measured by real-time RT-PCR. Each bar represents the mean ± S.D. of three independent experiments. Indicated levels of statistical significance were; * p<0.05, ** p<0.01, *** p<0.001 as compared with the control or DMSO cultures.

Cell proliferation, differentiation, and survival can be regulated by ERK 1/2, p38, and AKT-mediated signaling pathways [28]. The inhibitory effects of PI3K (LY294002, 5 μM), ERK (U0126, 5 μM), p38 (SB203580, 10 μM) and JNK (SP600125, 5 μM) inhibitors on monocyte proliferation were studied by CCK-8 analysis. Our current study showed that a block in p38 MAPK-dependent cell death might contribute to the increase in 20 μg/ml MBL-mediated monocyte proliferation, which was observed in the presence of SB203580 (Figure. 7A). This observation indicated that p38 MAPK, but not PI3K, ERK and JNK, was involved in MBL-mediated monocyte proliferation. A role for the p38 MAPK pathway has previously been identified using SB203580 in diverse cellular processes such as IL-2- and IL-7-mediated T cell proliferation [29].

We also investigated the intracellular signaling pathways that participated in MBL-mediated cell cycle arrest in monocytes. The effects of PI3K and MAPK inhibitors on cell cycle regulatory gene expression were analyzed by real-time RT-PCR. SB203580 suppressed the increase in p21 mRNA expression that was associated with MBL (Figure. 7B). Additionally, SB203580 resulted in an increased trend in cyclin D1 and Cdk4 mRNA expression that was associated with stimulation by MBL (Figure. 7C and 7D). It was the inhibition of p38 activation which correlated with cell survival (Figure. 7A), reduction of p21 (Figure. 7B) and increase of cyclin D1, Cdk4 (Figure. 7C and 7D). MBL-mediated activation of p38 signaling pathway promoted cell cycle arrest in monocyte and reduced cell viability.

## Discussion

This study presented data supporting a novel role for the innate immune protein MBL during the inhibitory effect on monocytes proliferation (Figure. 2). The effects of MBL on proliferation of cultured cells showed diverse effects distinctly from previously published studies with different sources of cells. For example, Ma et al. [30] found that MBL recognizes and specifically binds to oligosaccharide ligands expressed on the surfaces of a human colorectal carcinoma, and interestingly possessed a potent growth inhibiting activity against human colorectal carcinoma cells. This anti-proliferative effect indicated the potential effect of MBL in the prevention and treatment of cancer. For this reason, it may be meaningful to better understand the regulatory activity of MBL on the proliferation of monocytes, and comprehensively evaluate the effect over a wide concentration range of MBL.

On the one hand, the mechanism underlying the anti-proliferative effect of MBL appeared to be related to the induction of cell cycle arrest in the G0/G1 phase of the cell cycle (Figure. 3). The regulation of the expression of checkpoint proteins corresponding to the dynamics of the cell cycle also supported the cell cycle arrest that was mediated by MBL. By contrast, the anti-proliferative effect of MBL could be partly accounted for by apoptosis including the activation of the caspase cascade and subsequent PARP cleavage (Figure. 4 and Figure. 5). Moreover MBL increased the level of pro-apoptotic Bax in monocytes but decreased in U937 cells, and significantly decreased the level of anti-apoptotic Bcl-2 in monocytes and U937 cells, while markedly induced Fas was far greater in U937 cells than monocytes. It was clear that the different cells such as monocytes and U937 cells were the possibly reason of the no-consistent results.

The results of the current study indicated that higher levels of MBL induced apoptosis, and did so in a dose- and time-dependent manner in the U937 human monocyte cell-line. In addition, Hong et al. [31] showed that exposure of prostate cancer cells to C1q, a component like MBL, induced apoptosis. C1q has also been shown to attenuate microglial proliferation, increase production of reactive oxygen species and nitric oxide and trigger a transient calcium increase [32]. However, this occurs only in certain pathological conditions, such as in Alzheimer's disease where there is an impairment in the blood–brain-barrier, and the amyloid protein in the brain recruits microglia that bind to C1q, are activated, and thus cause deposition of amyloid plaques and tangles.

We have demonstrated that TGF-β1 mediated dampened cell proliferation by showing that SB-431542 almost completely abrogated growth inhibition due to MBL treatment of cells. We also showed that TGF-β1 secretion by monocytes could mediate such a change, and confirmed this observation using anti-TGF-β1, which reversed the inhibition of cell growth (Figure 6). Others have previously shown that recombinant TGF-β1 inhibits the proliferation and promotes differentiation of human promonocytic leukemic cells to macrophages [33]. Although activation may occur through various mechanisms upon cell contact, latent TGF-β may be activated by αV integrins, which may drive Smad signaling through a variety of pathways, including those transduced by TGF-β R or PI3K/Akt and MAPK. At higher concentrations, MBL inhibited M-CSF-induced monocyte proliferation, and did so through both TGF-β1 and p38 MAPK-dependent signaling pathways. These observations suggested that MBL could markedly blunt an inflammatory condition.

Monocytes and macrophages often function as immunological control switches in balancing the effects of pro- and anti-inflammatory reactions. Although monocytes represent an important component of host defense, accumulation of monocytes can be harmful and aggravate diseases such as atherosclerosis, arthritis and multiple sclerosis [34]. In the present study, higher doses of MBL inhibited monocyte proliferation, and did so by up-regulating cell cycle inhibitors, and pro-apoptotic signals. In addition, accumulating evidence suggests that apoptotic cells can actively suppress inflammation. That is, apoptotic cells inhibit the production of inflammatory mediators, and promote secretion of anti-inflammatory and immunoregulatory cytokines such as IL-10 by monocytes, macrophages, and DCs [35]. Thus, in many ways, our data support an important role for MBL in the regulation of adaptive immunity and inflammatory responses. Additionally, higher concentrations of MBL could inhibit LPS-induced DC maturation, and the secretion of pro-inflammatory cytokines like TNF-α and IL-12, and reduce the ability of antigen presenting cells (APCs) to stimulate allogeneic T lymphocyte proliferation [11]. These observations indicate that MBL could be implicated in both the anti-inflammatory effect and immunoregulation.

The concentration of MBL in the serum varies considerably among individuals depending on genetic polymorphisms, and normally ranges from 0.01 to 10 μg/ml [36]. However, as an acute-phase protein, the presence of MBL can increase up to threefold under inflammatory conditions [37]. For instance, the serum levels of MBL in patients with rheumatic heart disease have been shown to increase significantly up to 14 μg/ml [38]. We found that circulating MBL levels in type-2 diabetic patients were significantly higher than those of healthy people (Figure. S2). The observation seems to be concordant with recent articles describing increased MBL concentrations in patients presenting with type-1 diabetes with microvascular complications [39]. We also found that mRNA expression of Fas, caspase-3, and p21 by monocytes in type-2 diabetic patients were significantly higher than those found in healthy people. However, mRNA expression of cyclin D1, Cdk2, and Cdk4 were markedly downregulated in type-2 diabetic patients, indicating that high concentrations of MBL in type-2 diabetic patients might participate in the inhibition of monocyte proliferation.

Thus, by analogy, we presume that further studies could provide evidence that during inflammation, higher concentrations of MBL in the serum could be secreted, and do so to affect peripheral, local and extravascular tissue immune regulation. Additionally, patients presenting with type-2 diabetes are characterized by a state of chronic low-grade inflammation. In type-2 diabetes we currently do not know whether MBL plays a protective role in the pathogenesis of this condition, or the progression of the disease. By contrast, it has been suggested that MBL plays an unfavorable role in diabetic nephropathy. Thus additional studies are urgently required to determine whether there is a specific role for MBL as a therapeutic option in type-2 diabetes.

## Conclusions

We have obtained compelling evidence to suggest that high levels of MBL significantly inhibited monocyte proliferation by affecting both cell cycle dynamics and apoptosis. Our study has demonstrated a novel biological function of MBL in regulating monocyte proliferation. This observation provides evidence that MBL influences the immune system by inhibiting monocyte proliferation in addition to its established role as an opsonin. Finally, it would be tempting to speculate that high serological levels of MBL may promote anti-inflammatory effects.

## Supporting Information

Figure S1 **Purification of MBL and monocytes.** MBL was purified from pooled human plasma samples. SDS-PAGE and western immunoblot analysis showed that highly purified MBL was a functional multimer composed of 30KD peptide chains (A). It was highly bioactive, as demonstrated by a ligand-binding assay (data not shown). The cell preparations were incubated with human FITC-CD14 antibody and then analyzed by flow cytometry using the FACSCalibur. We found that more than 95% of the cells in each preparation were monocytes (B).
(TIF)

Figure S2 **Comparison of serum MBL levels and gene expression of monocytes in type 2 diabetic patients and healthy control subjects.** Distribution of serum MBL levels in healthy control subjects (•) and type 2 diabetic patients (▴) (A). The mRNA expression levels of cell cycle regulatory proteins and apoptosis-related proteins in monocytes are shown (B). Monocytes were isolated from healthy control subjects (n = 6) and type 2 diabetic patients (n = 6), and the mRNA expression levels of Fas, caspase-3, cyclinD1, Cdk2, Cdk4, and p21 in monocytes were analyzed by real-time RT-PCR. Horizontal bars represent medians within each group. Levels of statistical significance refer to the Mann-Whitney U test for differences between groups: * p<0.05, ** p<0.01, ***p<0.001 as compared healthy control subjects.
(TIF)

Table S1 **List of the sequences of primer for real-time PCR.** The primer sequences of different genes were listed as above. Forward was the forward primer and reverse was reverse primer nucleotide sequences, respectively.
(DOC)
