# Supplementary figures and images for: Mannose-Binding Lectin Inhibits Monocyte Proliferation through Transforming Growth Factor-β1 and p38 Signaling Pathways
Source: PLoS One. 2013 Sep 6;8(9):e72505. doi: 10.1371/journal.pone.0072505 (PMC3765169; doi:10.1371/journal.pone.0072505)

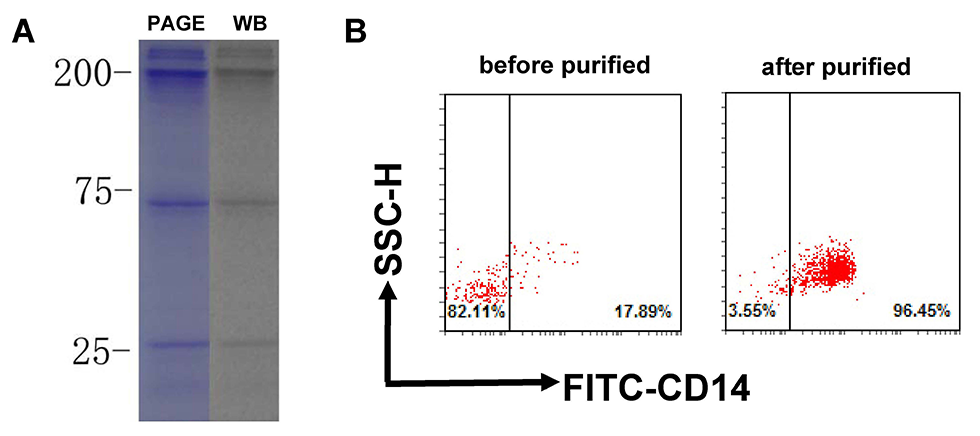

Supplement: Figure S1 — Purification of MBL and monocytes. MBL was purified from pooled human plasma samples. SDS-PAGE and western immunoblot analysis showed that highly purified MBL was a functional multimer composed of 30KD peptide chains (A). It was highly bioactive, as demonstrated by a ligand-binding assay (data not shown). The cell preparations were incubated with human FITC-CD14 antibody and then analyzed by flow cytometry using the FACSCalibur. We found that more than 95% of the cells in each preparation were monocytes (B). (TIF) [file pone.0072505.s001.tif]

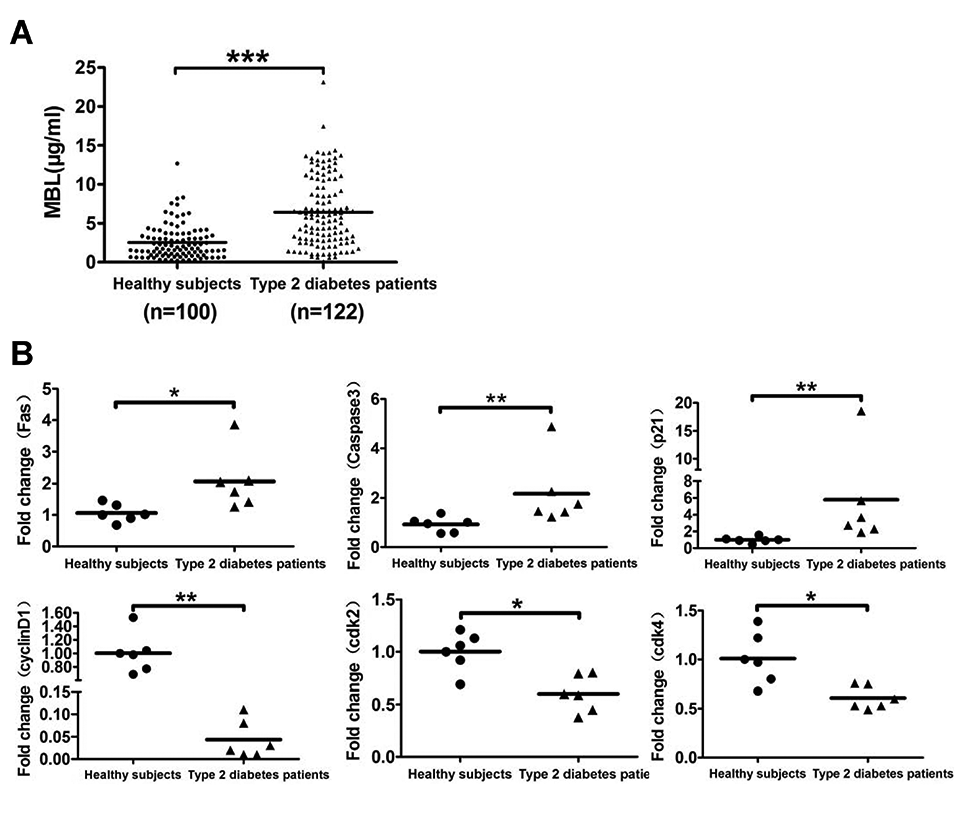

Supplement: Figure S2 — Comparison of serum MBL levels and gene expression of monocytes in type 2 diabetic patients and healthy control subjects. Distribution of serum MBL levels in healthy control subjects (•) and type 2 diabetic patients (▴) (A). The mRNA expression levels of cell cycle regulatory proteins and apoptosis-related proteins in monocytes are shown (B). Monocytes were isolated from healthy control subjects (n = 6) and type 2 diabetic patients (n = 6), and the mRNA expression levels of Fas, caspase-3, cyclinD1, Cdk2, Cdk4, and p21 in monocytes were analyzed by real-time RT-PCR. Horizontal bars represent medians within each group. Levels of statistical significance refer to the Mann-Whitney U test for differences between groups: * p<0.05, ** p<0.01, ***p<0.001 as compared healthy control subjects. (TIF) [file pone.0072505.s002.tif]
