# Supplementary material for: Mannose-Binding Lectin Inhibits Monocyte Proliferation through Transforming Growth Factor-β1 and p38 Signaling Pathways
Source: PLoS One. 2013 Sep 6;8(9):e72505. doi: 10.1371/journal.pone.0072505 (PMC3765169; doi:10.1371/journal.pone.0072505)
Supplement: Table S1 — List of the sequences of primer for real-time PCR. The primer sequences of different genes were listed as above. Forward was the forward primer and reverse was reverse primer nucleotide sequences, respectively. (DOC) [file pone.0072505.s003.doc]

**Table S1. List of the sequences of primer for real-time PCR**

| Genes | Human |
| --- | --- |
| Cyclin D1 | Forward: ATC TAC ACC GAC AAC TCC ATC |
|  | Reverse: TGT TCT CCT CCG CCT CTG |
| Cyclin D3 | Forward: CTG GAT GCT GGA GGT ATG TG |
|  | Reverse: GGA CGC AAG ACA GGT AGC |
| Cdk2 | Forward: AGT TAC TTC TAT GCC TGA TTA C |
|  | Reverse: TAC TGG CTT GGT CAC ATC |
| Cdk4 | Forward: CAC ATC CCG AAC TGA CCG |
|  | Reverse: AAT TGG CAT GAA GGA AAT CTA G |
| Cip1/p21 | Forward: CCT GTC ACT GTC TTG TAC C |
|  | Reverse: AAT CTG TCA TGC TGG TCT G |
| Kip1/p27 | Forward: GGC TAA CTC TGA GGA CAC |
|  | Reverse: GTA GAA GAA TCG TCG GTT G |
| Bax | Forward: AGG ATC GAG CAG GGC GAA TG |
|  | Reverse: CTG TGT CCA CGG CGG CAA T |
| Bcl-2 | Forward: CAA CAT CGC CCT GTG GAT GAC |
|  | Reverse: AGA GAC AGC CAG GAG AAA TCA AAC |
| Fas | Forward: CCA ATT CTG CCA TAA GCC CTG TC |
|  | Reverse: TTG GTA TTC TGG GTC CGG GTG |
| FasL | Forward: AGT TCT TCC CTG TCC AAC CTC TGT G |
|  | Reverse: CCT GTG CTG TGG TTC CCT CTC |
| Caspase-3 | Forward: CAA ACT TTT TCA GAG GGG ATC G |
|  | Reverse: GCA TAC TGT TTC AGC ATG GCA C |
| TGF-β1 | Forward: GCC CTG GAC ACC AAC TAT TGC T |
|  | Reverse: AGG CTC CAA ATG TAG GGG CAG G |
| GAPDH | Forward: CTC CTC CTG TTC GAC AGT CAG C |
|  | Reverse: CCC AAT ACG ACC AAA TCC GTT |
